# Cytoarchitecture, probability maps, and functions of the human supplementary and pre-supplementary motor areas

**DOI:** 10.1007/s00429-018-1738-6

**Published:** 2018-09-05

**Authors:** Jianghai Ruan, Sebastian Bludau, Nicola Palomero-Gallagher, Svenja Caspers, Hartmut Mohlberg, Simon B. Eickhoff, Rüdiger J. Seitz, Katrin Amunts

**Affiliations:** 1 C. and O. Vogt Institute for Brain Research, Medical Faculty, University Hospital Düsseldorf, Heinrich Heine University Düsseldorf, Düsseldorf, Germany; 2 Department of Neurology, The Affiliated Hospital of Southwest Medical University, Luzhou, China; 3 Centre of Neurology and Neuropsychiatry, LVR-Klinikum Düsseldorf, Medical Faculty, Heinrich-Heine-University Düsseldorf, Düsseldorf, Germany; 4 Institute of Neuroscience and Medicine (INM-1), Research Centre Jülich, Jülich, Germany; 5 Department of Psychiatry, Psychotherapy, and Psychosomatics, Medical Faculty, RWTH Aachen, and JARA Translational Brain Medicine, Aachen, Germany; 6 Institute for Systems Neuroscience, Medical Faculty, Heinrich-Heine University Düsseldorf, Düsseldorf, Germany; 7 Institute for Neuroscience and Medicine (INM-7), Research Centre Jülich, Jülich, Germany; 8 Department of Neurology, Medical Faculty, University Hospital Düsseldorf, Düsseldorf, Germany; 9 Florey Neuroscience Institutes, Melbourne, VIC Australia

**Keywords:** Supplementary motor area, Pre-supplementary motor area, Cytoarchitectonic mapping, Quantitative cytoarchitectonics, Probability maps, Meta-analytic connectivity modeling

## Abstract

The dorsal  mesial frontal cortex contains the supplementary motor area (SMA) and the pre-supplementary motor area (pre-SMA), which play an important role in action and cognition. Evidence from cytoarchitectonic, stimulation, and functional studies suggests structural and functional divergence between the two subregions. However, a microstructural map of these areas obtained in a representative sample of brains in a stereotaxic reference space is still lacking. In the present study we show that the dorsal mesial frontal motor cortex comprises two microstructurally different brain regions: area SMA and area pre-SMA. Area-specific cytoarchitectonic patterns were studied in serial histological sections stained for cell bodies of ten human postmortem brains. Borders of the two cortical areas were identified using image analysis and statistical features. The 3D reconstructed areas were transferred to a common reference space, and probabilistic maps were calculated by superimposing the individual maps. A coordinate-based meta-analysis of functional imaging data was subsequently performed using the two probabilistic maps as microstructurally defined seed regions. It revealed that areas SMA and pre-SMA were strongly co-activated with areas in precentral, supramarginal and superior frontal gyri, Rolandic operculum, thalamus, putamen and cerebellum. Both areas were related to motor functions, but area pre-SMA was involved in more complex processes such as learning, cognitive processes and perception. The here described subsequent analyses led to converging evidence supporting the microstructural, and functional segregation of areas SMA and pre-SMA, and maps will be made available to the scientific community to further elucidate the microstructural substrates of motor and cognitive control.

**Electronic supplementary material:**

The online version of this article (10.1007/s00429-018-1738-6) contains supplementary material, which is available to authorized users.

## Introduction

**Fig. 1:**
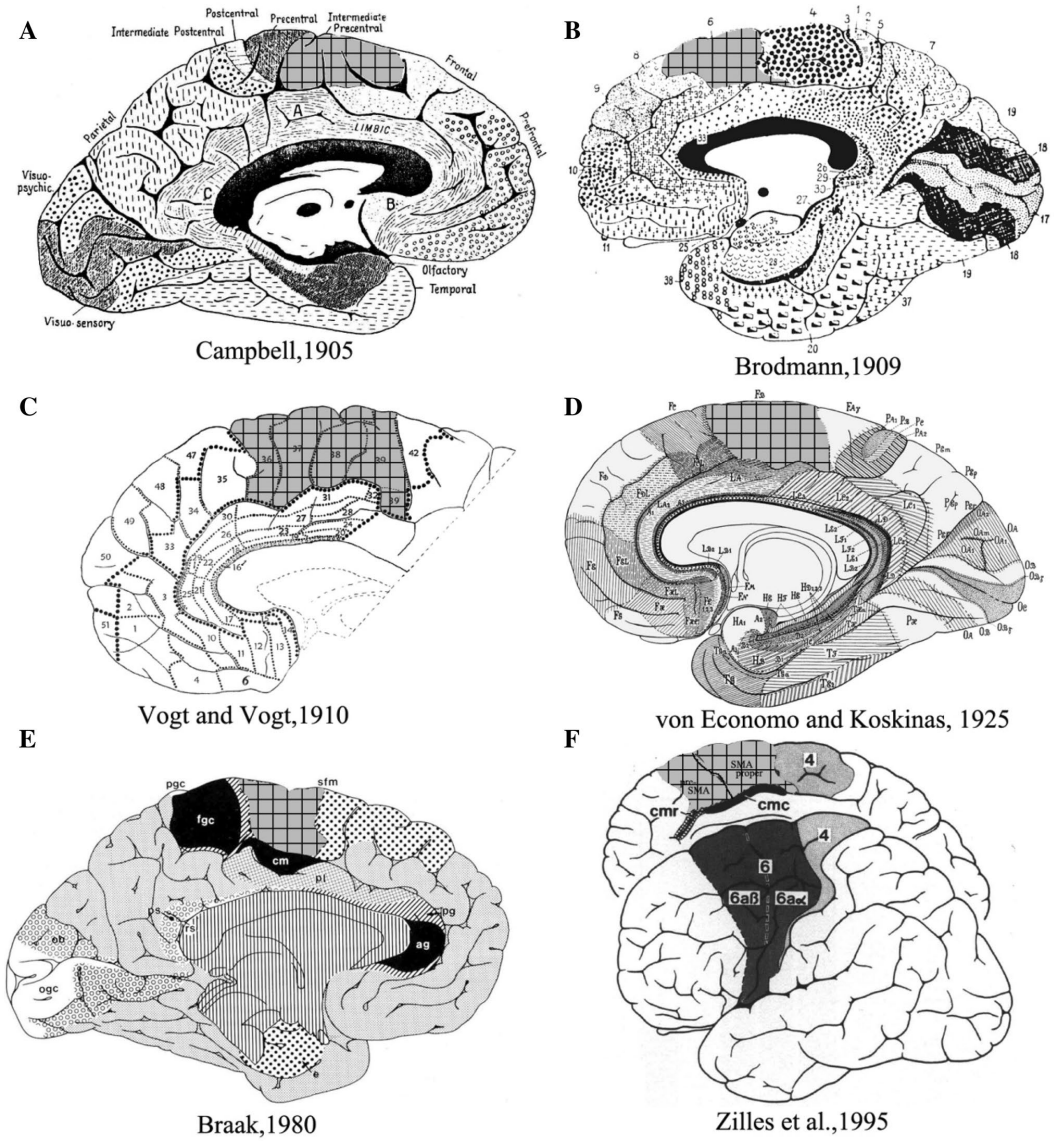
Published maps of the human mesial frontal motor cortex. The putative homologs with the mesial portion is Brodmann’s area 6 are highlighted in grid. **a** Campbell  (1905), **b** Brodmann (1909), **c** Vogt (1910), **d** von Economo and Koskinas (1925), **e** Braak (1980), **f** Zilles et al. (1995). The maps in **a, c, e** are based on myeloarchitectonic studies; Those in **b, d** are based on cytoarchitectonic studies; that in **f** on a combined cyto- and myelo- architectonic analysis

The human mesial frontal motor area has for a long time been thought of as being one entity. Campbell (1905) identified an intermediate precentral field, rostrally adjacent to the primary motor cortex based on cyto- and myeloarchitectonic criteria. These observations were later confirmed by Brodmann, who recognized a comparable region as the mesial part of his area 6 (Brodmann area, BA6; Brodmann 1909). Vogt and Vogt (1910) published the first detailed myeloarchitectonic map of the human frontal cortex, of which areas 36–41 were comparable to Brodmann’s area 6 (Vogt 1910). In 1925, von Economo and Koskinas identified their mesial frontal motor area FB, and described verbally and pictorially the variations in cellular structure of cerebral cortical layers (von Economo and Koskinas 1925; Triarhou 2007). Braak (1980) recognized a similar area as his superofrontal magnopyramidal region (sfm) (Braak 1980) (Fig. 1). Later cytoarchitectonic and functional neuroimaging studies identified the supplementary motor area (SMA or SMA proper) and the pre-supplementary motor area (pre-SMA) in the dorsal  mesial frontal motor region based on cytoarchitecture and receptor autoradiography (Zilles et al. 1995; Picard and Strick 1996). It has to be emphasized, however, that these maps differ regarding the number of subareas identified, as well as the relative size and extent of the areas SMA and pre-SMA.

The question if and how the mesial frontal cortex in the superior frontal gyrus is parcellated into functional units or modules has been a matter of interest in the past decades (Seitz et al. 2006). Importantly, it has been reported that areas SMA and pre-SMA can be defined by means of diffusion-weighted imaging (DWI) and subsequent probabilistic tractography. In this approach areas were defined based on the assumption that similar connectivity profiles could be found within a region (Johansen-Berg et al. 2004; Klein et al. 2007). More recent hypotheses emphasize parcellations based on functional connectivity, e.g., a definition of the putative areas SMA and pre-SMA using resting state fMRI (Kim et al. 2010; Joliot et al. 2015). Another approach identified areas SMA and pre-SMA in a data-driven fashion based on their individual co-activation patterns (Eickhoff et al. 2011). These clustering solutions of the mesial frontal cortex are biologically meaningful considering that the region of interest may show a multi-layered functional hierarchy (Geyer and Turner 2015).

Areas SMA and pre-SMA are involved in a variety of functions related with motor sequence processing, planning and executing (Hoshi and Tanji 2004; Cona et al. 2016; Hupfeld et al. 2016), as well as temporal and spatial processing of movements (Mita et al. 2009; Kotz and Schwartze 2011). Furthermore, pre-SMA and SMA also contribute to motor learning, speech and language processing (Seitz et al. 2006; Kim and Shin 2014; Hertrich et al. 2016). Interestingly, pre-SMA and SMA also mediate motor inhibition (Toma et al. 1999; Li et al. 2006). But important functional differences have been identified between these two areas. For example, the selection of an intended action, and the expected reward thereof have been reported to modulate neural activity in the SMA (Elliott et al. 2003; Lee 2004). Conversely, the pre-SMA has been considered as a motor control area guiding movement selection and scaling (Nachev et al. 2008; Ogawa et al. 2006) and been implicated even in non-motor functions such as empathy (Seitz et al. 2008). Furthermore, the pre-SMA is considered the most likely generator of the so-called Bereitschaftspotential, which occurs as early as 1000 ms before movement onset (Kornhuber and Deecke 1965; Lau and Passingham 2007; Iacoboni et al. 2005). It is, therefore, reasonable to expect that these two areas can be differentiated in terms of microanatomy and functional connectivity patterns.

The aim of the present study was to explore the cytoarchitectonical and functional dichotomy of areas SMA and pre-SMA. For that purpose, we applied a novel approach for anatomical and functional characterization of human brain areas. It should be noted that the cytoarchitectonic and receptor autoradiography maps in the work by Zilles et al. (1995) determined by observer dependent descriptions, were available only as two-dimensional schematic drawings and did not include inter-individual variability of different brains. Therefore, the data could not be matched with functional imaging data. In this study we attempted to overcome these limitations. We acquired 3D datasets, as we first analyzed the cytoarchitecture of the human mesial frontal motor area in serial histological sections of ten human postmortem brains and used a statistical approach for the observer-independent detection of borders between cortical areas (Schleicher et al. 2005, 2009). Second, the resulting individual maps of areas SMA and pre-SMA were then transferred to a common stereotaxic reference space to calculate continuous probabilistic maps, which quantify inter-subject variability in the localization and extent of areas SMA and pre-SMA, and maximum probability maps. Third, these maximum probability maps of areas SMA and pre-SMA served as seed volumes of interest for a coordinate-based meta-analysis to investigate the functional co-activation patterns of each area.

## Materials and methods

### Histological processing of postmortem brains

Ten brains of subjects without a clinical history of neurological or psychiatric diseases in medical records (five females and five males; Table 1), were obtained through the body donor program of the Department of Anatomy at the University of Düsseldorf. The brains were extracted from the skull within 24 h after death and fixed in formalin or Bodian’s fixative for at least 6 months. Following fixation and removal of the meninges, each brain was subjected to magnetic resonance imaging (MRI) (1.5 T, Siemens, Germany) inspection in a T1-weighted 3D FLASH phase (flip angle 40°, reception time = 40 ms, time echo = 5 ms). Then, the whole brains were embedded in paraffin and serially cut in the coronal (*n* = 9) or horizontal plane (*n* = 1, case 17) on a large-scale microtome (Polycut E, Reichert-Jung, Germany; thickness = 20 µm). Each 15th section was mounted on a glass slide and stained for cell bodies using modified silver staining (Merker 1983).

**Table 1:** The postmortem brains obtained for cytoarchitectonic analysis

Brain	Gender	Age (year)	Cause of death
1	F	79	Bladder cancer
2	M	56	Rectal cancer
4	M	75	Necrotizing glomerulonephritis
6	M	54	Myocardial infarction
9	F	79	Generalized atherosclerosis
10	F	95	Small bowel obstruction
12	F	43	Pulmonary heart disease
17	F	50	Acute myocardial infarction
20	M	65	Congestive heart failure, respiratory failure
21	M	30	Bronchopneumonia, deep vein thrombosis

*F* female, *M* male

### Observer-independent detection of cytoarchitectonic borders

An observer-independent mapping approach (Schleicher et al. 2005) was used to identify cortical borders. This approach was built on the assumption that the laminar pattern is similar within a cytoarchitectonic area, but varies abruptly at the border between two adjacent areas. In practice, rectangular regions of interest (ROIs) were obtained with a high-resolution CCD-Camera (Axiocam MRM, ZEISS, Germany), which worked with an optical light microscope (Axio Observer Z1, ZEISS, Germany). ROIs were digitized with an in-plane resolution of 1.02 µm per pixel and transformed into grey-level index (GLI) images with in-house software written in MatLab (The MathWorks, Inc., Natick, MA, USA). These GLI images provide quantitative information about the volume fraction of cell bodies (Wree et al. 1982). For computation, the ROI was subdivided into measuring fields (17 × 17 pixels in size) by a grid, cell bodies were identified and segmented by adaptive thresholding, and all pixels in a measuring field which were labeled as cells were transferred to an 8 bit grey value which coded the volume fraction of stained cell bodies in that measuring field (Schleicher et al. 2005).

Equidistant GLI profiles were extracted between the outer contour line (the border between layers I and II) to the inner contour line (the border between layer VI and the white matter), which were manually traced on each high-resolution scanned image. A set of ten features (including the mean GLI value, the center of gravity in the axis of the cortical depth, the standard deviation, the skewness, the kurtosis, and the analogous parameters for the first derivative of each profile) was extracted from each GLI profile and used to quantify the laminar pattern of the cortical ribbon at that position (Schleicher et al. 2000, 2009).

**Fig. 2:**
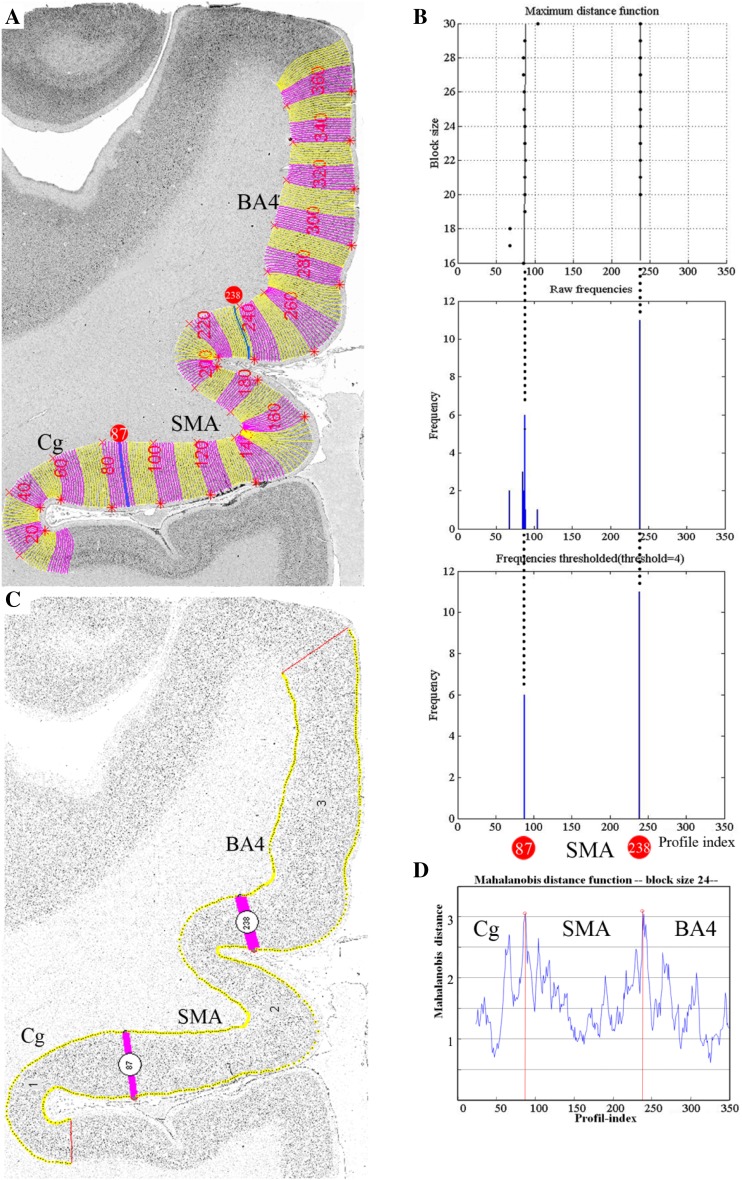
Observer-independent border detection. **a** Digitized ROI with contour lines and superimposed numbered curvilinear traverses indicating, where the GLI profiles were extracted. The bars at profile positions 87 and 238 corresponds to the significant maxima of the Mahalanobis distance (MD) function in **d** caused by the cytoarchitectonic border between the cingulate cortex (Cg) and SMA, SMA and area 4 which has been confirmed by microscopical examination. (**b**, maximal distance function) Dependence of the position of significant maxima of the MD function (black dots) with different block size. The result was Bonferroni corrected (*P* = 0.001). **b** The corresponding frequency of significant maxima at different maxima at different profile positions across block sizes 12–30. **c** The positions of significant maxima (i.e., cortical borders) detected by an in-house software written in MatLab. **d** Single MD function at block size 24 with a significant maximum at profile positions 87 and 238. *SMA* supplementary motor area, *Cg* cingulate cortex, *BA4* Brodmann’s area 4

The Mahalanobis distance *D*^2^ (Mahalanobis et al. 1949) was used to quantify differences between the feature vectors of adjacent groups of profiles (Schleicher et al. 2005). To decrease the noise during the calculation of the Mahalanobis distance, a specified number of profiles (ranging from 10 to 30 profiles) were grouped into a block of profiles and the *D*^2^ between adjacent blocks was computed. This procedure was repeated along the entire portion of the cortical ribbon covered by the ROI by means of a sliding window procedure shifting the blocks of profiles by one profile each time (Schleicher et al. 2000). The larger the difference between profiles of adjacent blocks, the larger the ensuing *D*^2^. A subsequent Hotelling’s t2 test with Bonferroni correction was selected to test for significant differences in *D*^2^ values, and the threshold was set at а = 0.001. Statistically significant maxima in the Mahalanobis plot were interpreted as indicating cortical borders. Borders between cytoarchitectonic areas were detected at positions with significant differences in laminar patterns at a minimum of [two] different block sizes and comparable positions in a minimum of three adjacent brain sections (Schleicher et al. 1999) (Fig. 2).

### Interareal differences in cytoarchitectonic profiles

To quantitatively detect the cortical layer(s) responsible for the cytoarchitectonic differences between areas SMA and pre-SMA, the mean GLI profiles of each area were divided into ten bins with a 10%-interval: bin 1 (0–10%), bin 2 (10–20%), …, bin 10 (90–100%). The mean GLI profile of each area in each brain was calculated from six samples (three samples per hemisphere and brain). In each sample, representative blocks of 15 GLI profiles were extracted, and mean GLI profiles produced. The mean GLI profiles of area SMA were then compared with those of area pre-SMA using pairwise permutation comparisons (*p* < 0.05). To avoid interferences caused by tangential sectioning, ROIs were collected at sites, where the cortex appeared to be cut perpendicular to the surface.

### Volumetric analysis

The percentage of area volume and the total brain volume were calculated to enable comparison of differently sized brains and to analyze individual variability in the size of SMA and pre-SMA. Furthermore, a shrinkage factor was defined for each postmortem brain as the ratio between the fresh volume and the histological volume to compensate for shrinkage during the histological processing (Amunts et al. 2007). We tested for differences of the volume proportion under the aspect of hemisphere and gender differences with a pairwise permutation test using in-house software written in Matlab (*p* < 0.05, false discovery rate corrected for multiple comparisons) (Bludau et al. 2014). The null distribution was evaluated by Monte Carlo simulation with a repetition of 1,000,000 iterations. Subsequently, correlation between volume and age was analyzed by computing the Spearman’s rank correlation coefficient (*p* < 0.05).

### Three-D reconstruction and probabilistic maps

The borders of areas SMA and pre-SMA were interactively transferred to high-resolution digitized images of the histological sections (1200 dpi; ~ 20 µm/pixel; 8 bit grey value resolution). Then, the mapped areas were three-dimensionally (3D) reconstructed using the MR image sets of the ten corresponding postmortem brains. The individual brains and the delineations of the cortical areas were spatially normalized to the T1-weighted single-subject template of the Montreal Neurological Institute (MNI) (Evans et al. 1992, 2012) reference brain with linear and non-linear transformation tools (Henn et al. 1997; Hömke 2006). Having all brains and delineations in the same reference space allowed us to calculate continuous probabilistic maps for each area in MNI space. The probabilistic map of a given area represents for each voxel of this particular area how many individuals showed the respective cytoarchitectonic area in that voxel. Thus, these maps quantitatively describe the inter-subject variability of a cortical area in stereotaxic space.

Subsequently, a maximum probability map (MPM) of the individual probabilistic maps were produced (Eickhoff et al. 2005, 2006). Within the MPM, each voxel was assigned to the cytoarchitectonic area with the most likely anatomical probability at that position. These MPMs of areas SMA and pre-SMA were used to define the volumes of interest in the coordinate-based meta-analysis.

### Meta-analytic connectivity modeling

To explore the area-specific functionality of SMA and pre-SMA, a coordinate-based meta-analysis was performed on the co-activation maps of the two selected seed regions in task-based activation data (Eickhoff et al. 2010). This meta-analytic connectivity modeling (MACM) employed the revised activation likelihood estimation (ALE) technique applied to the BrainMap database (http://brainmap.org/) (Laird et al. 2005, 2011). The main principle of the ALE method is to treat the reported foci as centers for 3D Gaussian probability distributions to capture the spatial uncertainty related with each focus. All reported foci within a 5 mm radius of the seed regions were identified (Eickhoff et al. 2011). This is a moderate compromise to reduce image noise over false negatives. To recognize random and non-random foci of convergence, the obtained ALE values were compared with a null distribution reflecting a random spatial correlation between the considered experiments with familywise error (FWE) corrected *p* threshold of *p* < 0.05 (Eickhoff et al. 2012).

The seed regions were defined by the former created MPMs of areas SMA and pre-SMA. Only those experiments were included, that reported stereotaxic coordinates from normal individual mapping studies in healthy humans using fMRI or PET. Accordingly, 1398 functional imaging data of pre-SMA (18,574 subjects, 21,552 foci) and 597 functional imaging data of SMA (7947 subjects, 9112 foci) were included. The analysis set a threshold at a familywise error (FWE) corrected cluster-level (*p* < 0.05).

To facilitate the functional interpretation of the co-activations patterns provided by the meta-analysis, the “Behavioral Domain” and “Paradigm Class” were categorized by several hierarchically structured keywords as given in the original articles present in the BrainMap database. “Behavioral Domain” classified the research in terms of the neural systems studied according to six main categories and related subcategories: cognition, action, perception, emotion, interoception, or pharmacology. “Paradigm Class” categorized tasks in the jargon of the field, such as anti-saccades or mental rotation tasks (Laird et al. 2009). Note, that the categories did not involve a post-hoc catergorization of the motor tasks in the original studies nor of limb versus eye movements or speech production.

## Results

### Identification of areas SMA and pre-SMA

**Fig. 3:**
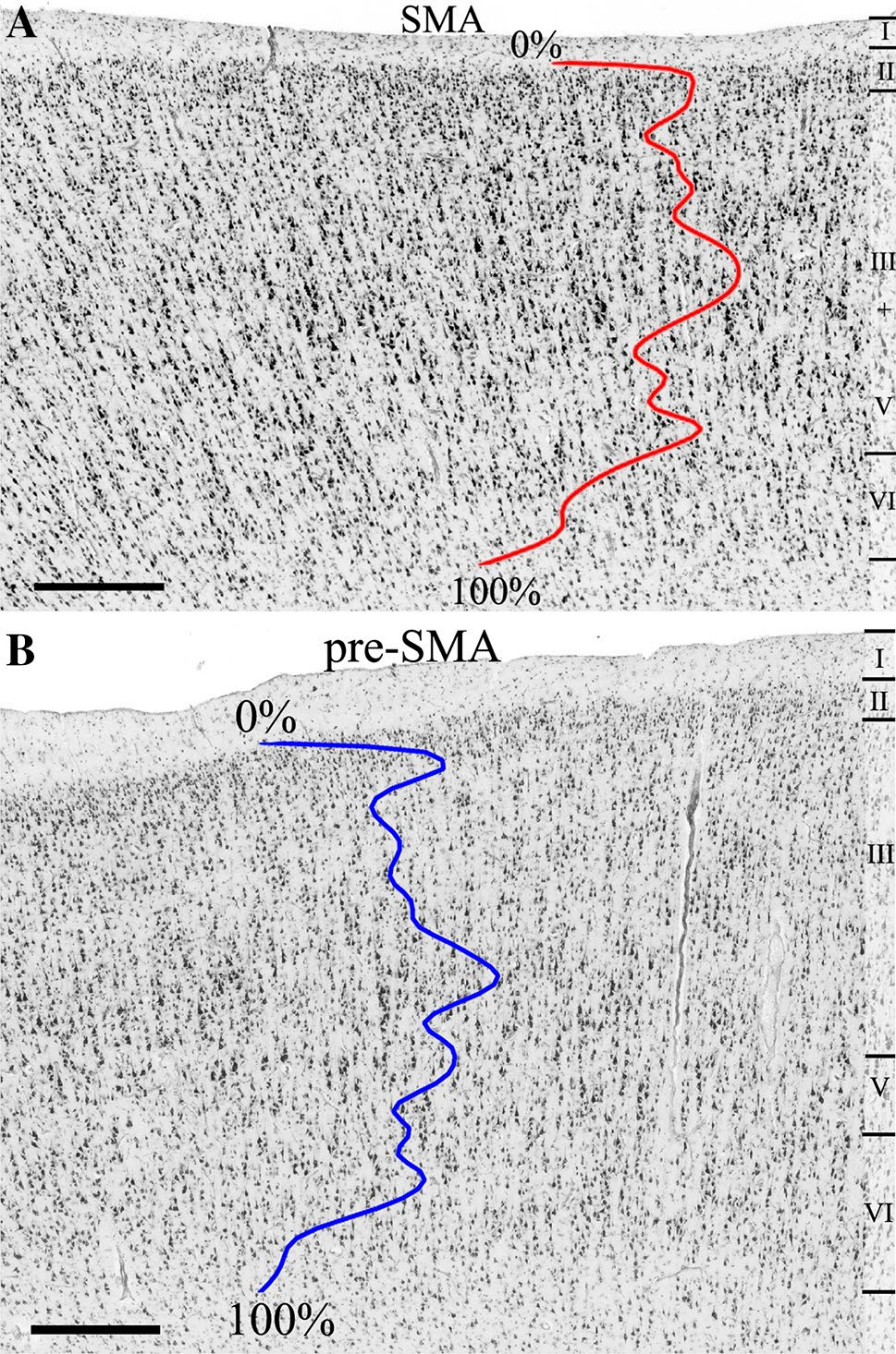
Cytoarchitecture of areas SMA (**a**) and pre-SMA (**b**) in horizontal, cell-body-stained sections (brain code 17, right hemisphere). The colored lines code the mean GLI profiles of areas SMA and pre-SMA. Roman numerals indicate the different cortical layers. Scale bars 0.5 mm

**Fig. 4:**
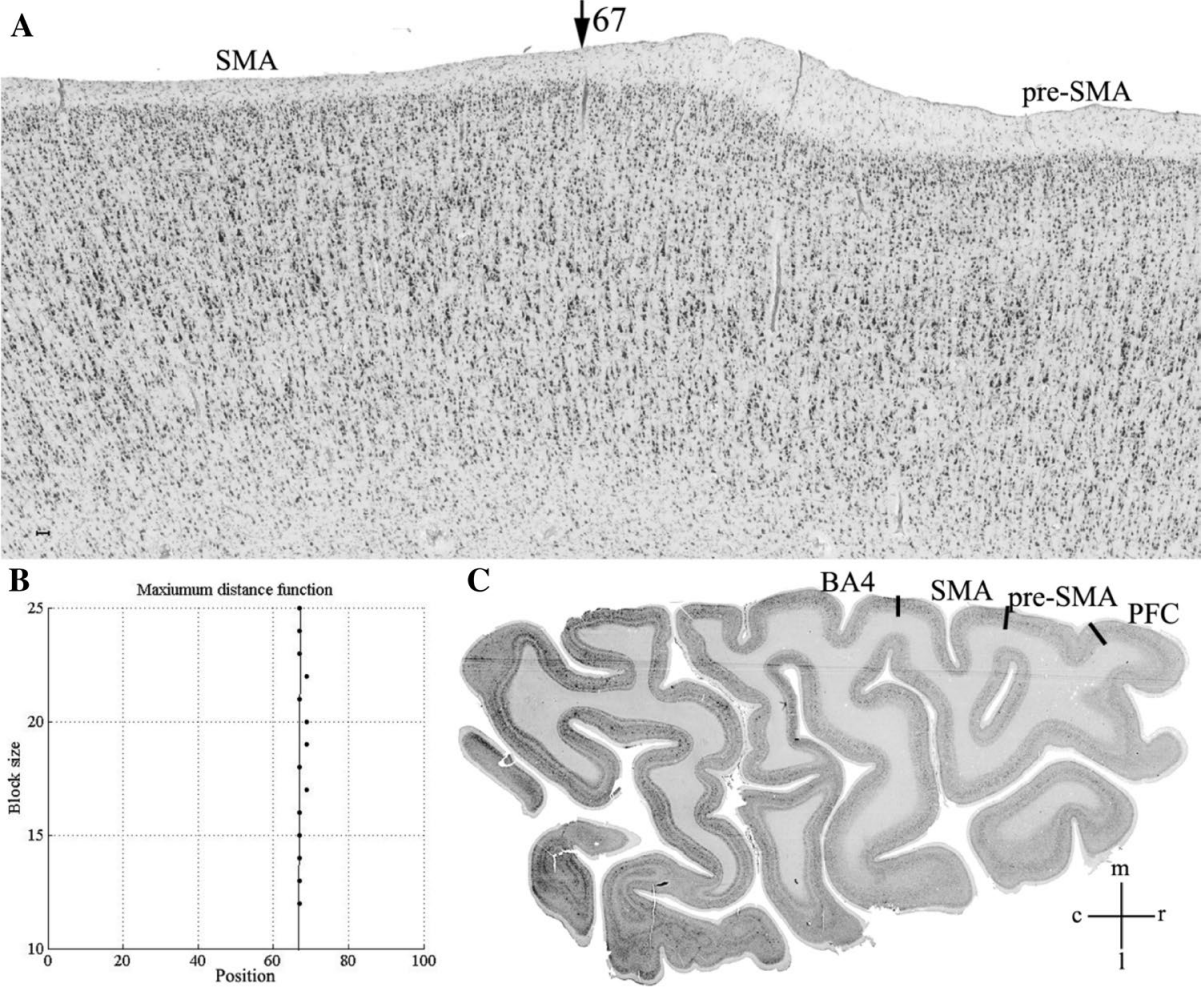
**a** Border between areas SMA and pre-SMA in a section of case 17 at position 67 (arrowhead) of the distance function. Horizontal, cell-body-stained section (the same place as in Fig. 5). **b** The position of the maximum of the Mahalanobis distance function *D*^2^ at position 67 (*p < 0.001) exactly matches that of the microscopically identified border. **c** Location of the region of interest in the right hemisphere. The dark lines between areas indicate borders identified by visual inspection. *BA4* primary motor area, *PFC* prefrontal cortex, *m* mesial, *l* lateral, *r* rostral, *c* caudal. Scale bars 0.5 mm

**Fig. 5:**
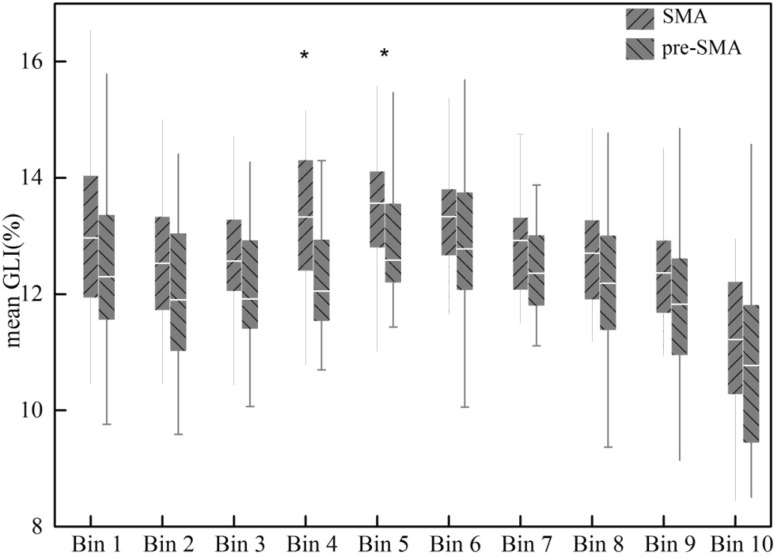
Box plots of the 10%-interval bins (*x* axis) into which the mean profiles (grey-level index, GLI, %, *y* axis) of areas SMA and pre-SMA were divided for statistical analysis. Bin 1, Bin 2,…Bin 10 indicate the bin from the border between layers I and II (Bin 1) to the border between layer VI and the white matter (Bin 10). Center horizontal indicated the mean GLI values of each bin in each area. *Bins 4 and bins 5 in the two groups showed significant differences (*p* < 0.05, pairwise comparison)

Two agranular areas, SMA and pre-SMA, were identified on the mesial surface of the hemisphere rostral to the primary motor cortex. Area SMA abutted the primary motor cortex and was followed rostrally by pre-SMA. Area SMA was characterized by a poor lamination, prominent large sized pyramidal cells in the lower part of layer III (i.e., layer IIIc), and the absence of Betz cells in layer V. Area pre-SMA differed essentially from area SMA by its dark layer V, which was well demarcated from layers III and VI. In SMA the boundary between layers III and V was not clearly visible. Layer III pyramids were clearly smaller in pre-SMA than in SMA (Table 2; Fig. 3). These differences in cytoarchitecture were reflected by differences in the GLI profiles of each area, with higher GLI values in the lower part of layer III of SMA than in that of pre-SMA (Fig. 3), thus enabling identification of the border between both areas with the observer-independent quantitative procedure (Fig. 4). Furthermore, statistical analysis of the ten bins into which the mean GLI profiles of each of the areas were divided revealed significant differences at the level of bin 4 and bin 5 (Fig. 5). The position of bins 4 and 5 coincided roughly with the location of layer IIIc, i.e., the layer with prominent large pyramidal cells in SMA, but less densely packed and with smaller pyramids in pre-SMA (Fig. 3).

**Table 2:** Criteria for identification of areas SMA and pre-SMA in  microscope inspection

Area	Criteria
SMA	1. Prominent large sized pyramidal cells in the lower part of layer III 2. Poor lamination, especially due to the fusion of layers IIIc and layer V 3. Occasional presence of big pyramidal cells in layer V, absence of Betz cells
Pre-SMA	1. Dark layer V well demarcated from layers III and VI 2. Clear lamination 3. Pyramidal cells located in layer IIIc are smaller than those in SMA

Areas SMA and pre-SMA were restricted to the medial surface of the hemisphere in each brain. Although their dorsal borders were often found close to the dorsal convexity of the hemisphere, they never encroached onto its lateral surface (Supplementary Fig. 1). The ventral borders of areas SMA and pre-SMA was found in the region of the cingulate sulcus. For both areas, the border was found on the dorsal bank of the sulcus, though that of pre-SMA was closer to the free surface of the hemisphere. There were no gyri or sulci reliably associated with the border between areas SMA and pre-SMA (Supplementary Fig. 1). However, the border between areas SMA and pre-SMA was closely associated with the vertical plane through the anterior commissure, i.e., the VCA line.

### Cytoarchitectonic borders with neighboring cortical areas

Areas SMA and pre-SMA were limited dorsally by the dorsal premotor cortex (PMd), i.e., by the portion of BA6 found on the lateral surface of the superior frontal gyrus. SMA mainly abuts the caudal part of PMd (PMdc), and pre-SMA its rostral part (PMdr). Ventrally, areas SMA and pre-SMA were followed by cingulate areas 24d and 24c, respectively (Palomero-Gallagher et al. 2018). The caudal border of SMA was with the primary motor cortex (BA4), and the rostral border of pre-SMA was with prefrontal cortex (specifically with BA8). The transition between BA4 and area SMA roughly coincided with the transition between cingulate areas 24d and BA23.

#### SMA and BA4

Both areas showed a poor lamination and a visible layer IV was absent, i.e., they are agranular. BA4 was characterized by the occurrence of giant pyramidal cells (Betz cells) in layer Vb. Unlike BA4, SMA showed no giant pyramidal cells in layer Vb, although some large pyramidal cells were found in its caudal part. Most importantly, SMA showed an increased cellular density in the lower part of layer III (Supplementary Fig. 2).

#### SMA and PMdc

Both areas were relatively poorly laminated, but had a conspicuous layer IIIc, which was denser and contained larger pyramids in PMdc than in SMA. Furthermore, layer VI of PMdc was more pronounced than that of SMA (Supplementary Fig. 3).

#### SMA and 24d

Both areas were agranular, but differed considerably in layers III and V. Area SMA, presented a prominent layer IIIc with medium size pyramids, whereas in area 24d cells in the lower part of layer III were considerably smaller. Moreover, whereas layer V is inconspicuous in SMA, a dark layer V with big or even giant pyramidal cells and well demarcated layer VI were easily identified in area 24d (Supplementary Fig. 4).

#### Pre-SMA and PMdr

Both pre-SMA and PMdr presented a relatively prominent layer V and a diffuse border between layers II and III. However, layer V neurons were larger and layer VI was more demarcated in PMdr than that in pre-SMA (Supplementary Fig. 5).

#### Pre-SMA and 24c

Both areas were characterized by a clear lamination and a relatively prominent layer V. However, layer V of 24c presented a higher packing density and larger cells than that of pre-SMA. Furthermore, the border between layers V and VI was sharper in 24c (Supplementary Fig. 6).

#### Pre-SMA and BA8

The border between these two areas was clearly identifiable, because layer IV was not visible in cell-body-stained sections of pre-SMA, while in BA8 this layer is clearly recognizable (Supplementary Fig. 7).

### Volumetric analysis

The average total volume proportions of left and right SMA, and left and right pre-SMA in relation to the whole brain were 1.3%, 1.27%, 1.51%, and 1.47%, respectively. No gender and hemispheric differences were found (Table 3). The correlation test showed no interaction between age and volume.

**Table 3:** Volume proportion analyses of area SMA and pre-SMA grouped by hemisphere and gender

Areas	Hemispheric differences			Gender differences			Hemispheric gender interaction		
	L	R	*p*	Female	Male	*p*	Female: L-R	Male: L-R	*p*
SMA	1.30 ± 0.14	1.27 ± 0.10	0.52	2.58 ± 0.18	2.55 ± 0.24	0.86	− 0.01 ± 0.16	0.06 ± 0.12	0.45
pre-SMA	1.51 ± 0.15	1.47 ± 0.14	0.49	3.02 ± 0.57	2.92 ± 0.58	0.74	0.06 ± 0.52	0.01 ± 0.36	0.68
Total	2.80 ± 0.46	2.73 ± 0.28	0.42	5.60 ± 0.75	5.47 ± 0.79	0.73	0.06 ± 0.67	0.08 ± 0.41	0.90

The data is represented as mean ± standard deviation

Significance threshold was set at *p* < 0.05

*Volume proportion* volume area/brain volume × 100%, *p* contrast estimate of the pairwise permutation tests, *L* left hemisphere, *R* right hemisphere, *Total* combined volume proportion s of SMA and pre-SMA

### Three-D probability maps and maximum probability maps

**Fig. 6:**
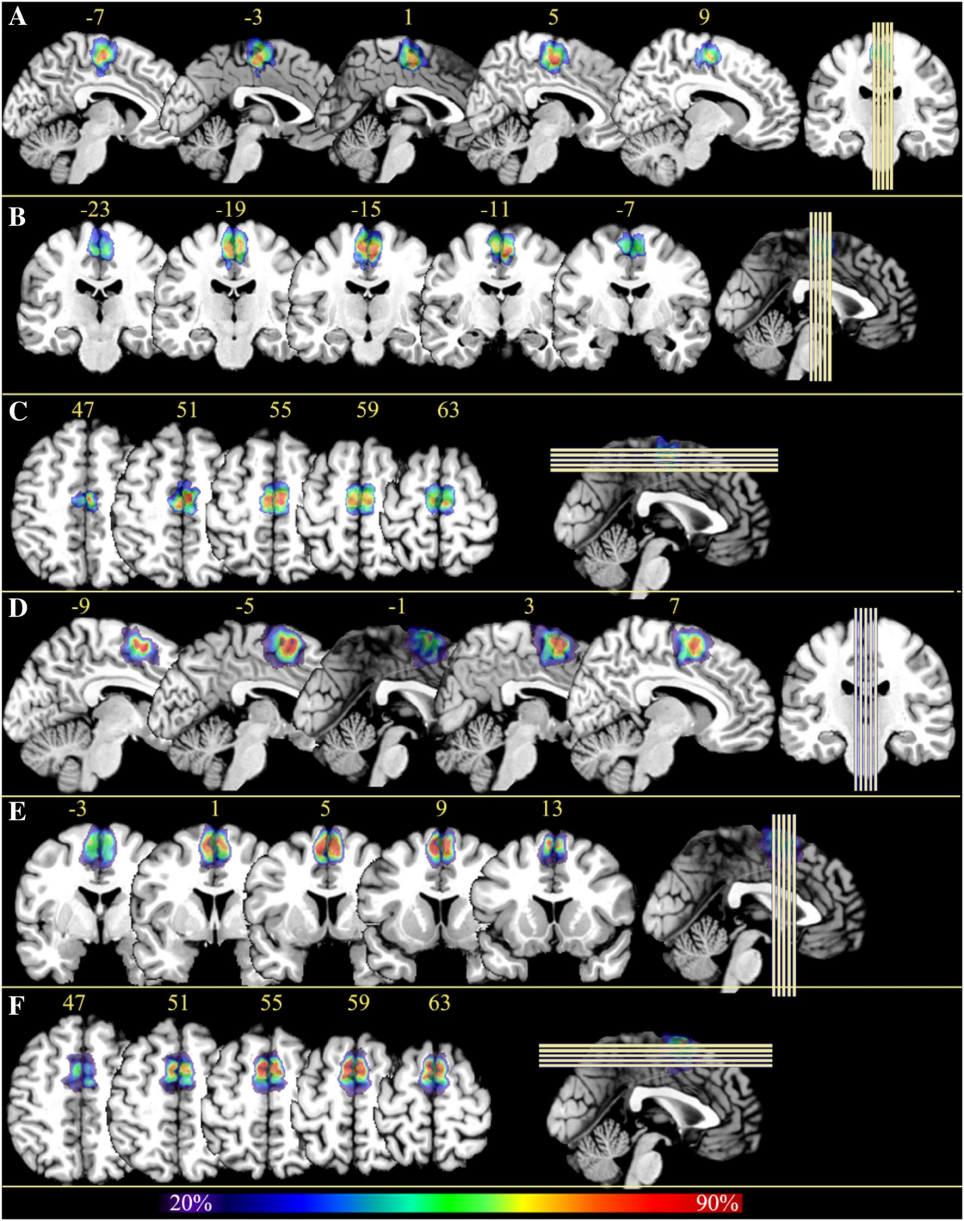
Continuous probability maps of areas SMA and pre-SMA in anatomical MNI reference brain. Cytoarchitectonic probability map of area SMA in the sagittal (**a**), coronal (**b**), horizontal (**c**) and sectioning planes, as well as of pre-SMA, also in the sagittal (**d**), coronal (**e**), horizontal (**f**), respectively. The number of overlapping brains for each voxel is color coded. The left and right side of each section in **b, c, e, f** indicate the left and right hemisphere, respectively. The yellow numbers in **a**–**f** indicate the *x*-, *y*-, *z*-coordinates, respectively. The images at the extreme right of panels indicate the locations of corresponding sections

Probabilistic cytoarchitectonic maps of areas SMA and pre-SMA were computed in the anatomical MNI reference brain (Amunts et al. 2005). The superimposition of all ten brains showed that areas SMA and pre-SMA are located dorsal to the cingulate sulcus in the mesial view. There was a relatively large inter-individual anatomical variability in the size of the areas, as indicated by the fact that voxels coding for a low overlap (blue and green tones) were more frequent than voxels coding for a high overlap (red and orange tones) (Fig. 6).

**Fig. 7:**
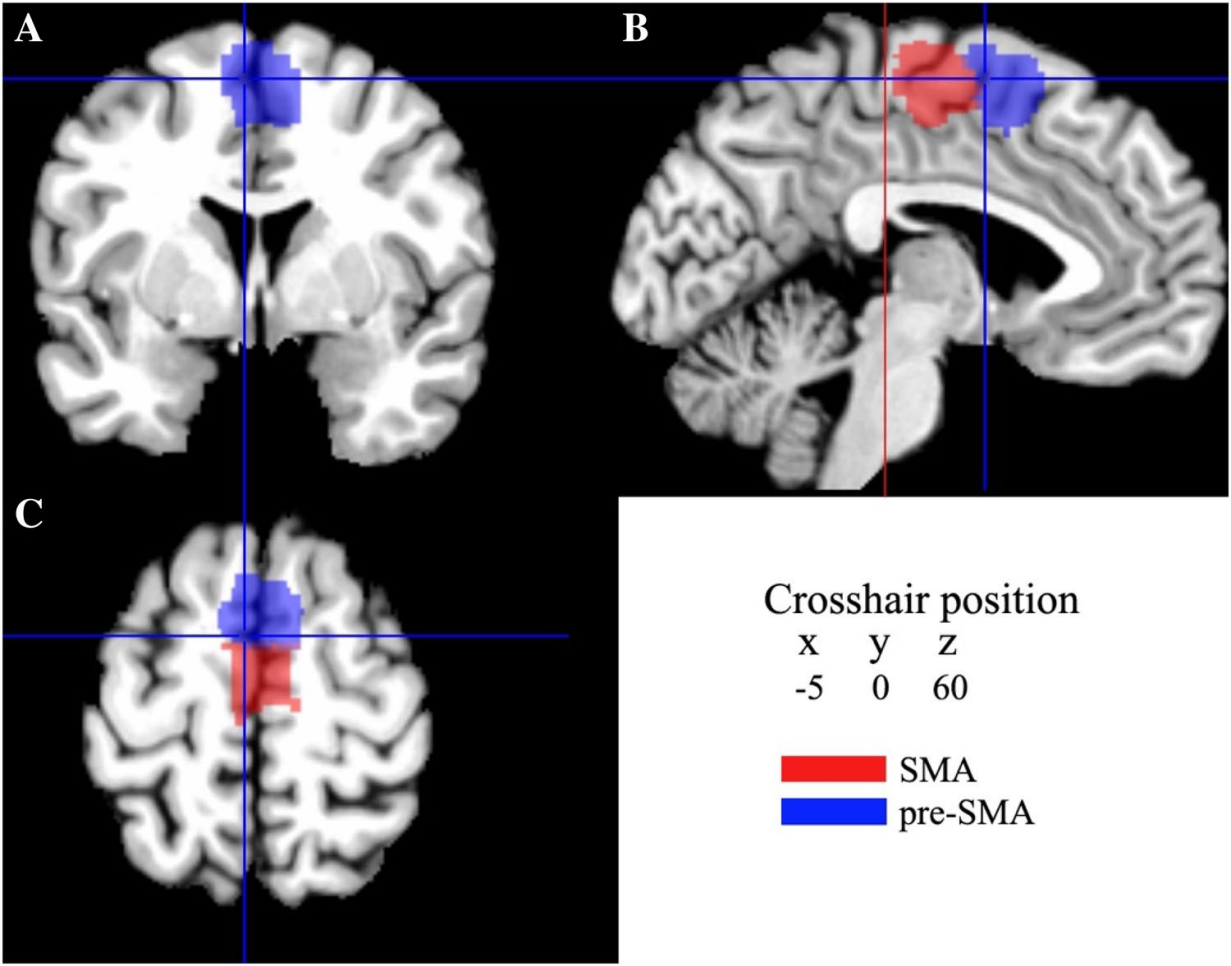
Sections through the MNI single-subject template showing the maximum probability maps of areas SMA and pre-SMA in the coronal (**a**), sagittal (**b**), and horizontal (**c**) planes of sectioning. The vertical axis of the crosshair in **b** crossed the anterior commissure (*y* = 0). The red line in **b** crossed the posterior commisure (*y* = − 28). The current version of the maximum probability maps was used for the analysis of structural magenetic resonance imaging data by means of the anatomy Tool box (Eickhoff et al. 2005), and can be downloaded at http://www.fz-juelich.de/inm/inm-1/spm_anatomy_toolbox

MPMs of areas SMA and pre-SMA were computed (Fig. 7). This non-overlapping parcellation of areas SMA and pre-SMA in a sample of ten human post mortem brains reflected the most likely voxels of the areas. The caudal boundary of area SMA was located anterior to the vertical plane through the posterior commissure (*y* = − 28); the border between areas SMA and pre-SMA was closely associated with the vertical plane through the anterior commissure (*y* = 0). The mean center of gravity of area SMA was caudal to the anterior commissure and that of the pre-SMA rostral to the anterior commissure in the anatomical MNI space (Table 4).

**Table 4:** Centers of gravity in anatomical MNI space of each area

Area	Center of gravity		
	*x*	*y*	*z*
Left SMA	− 5	− 10	54
Right SMA	4	− 11	53
Left pre-SMA	− 5	6	53
Right pre-SMA	4	6	53

### Coordinate-based meta-analysis of functional imaging studies

**Fig. 8:**
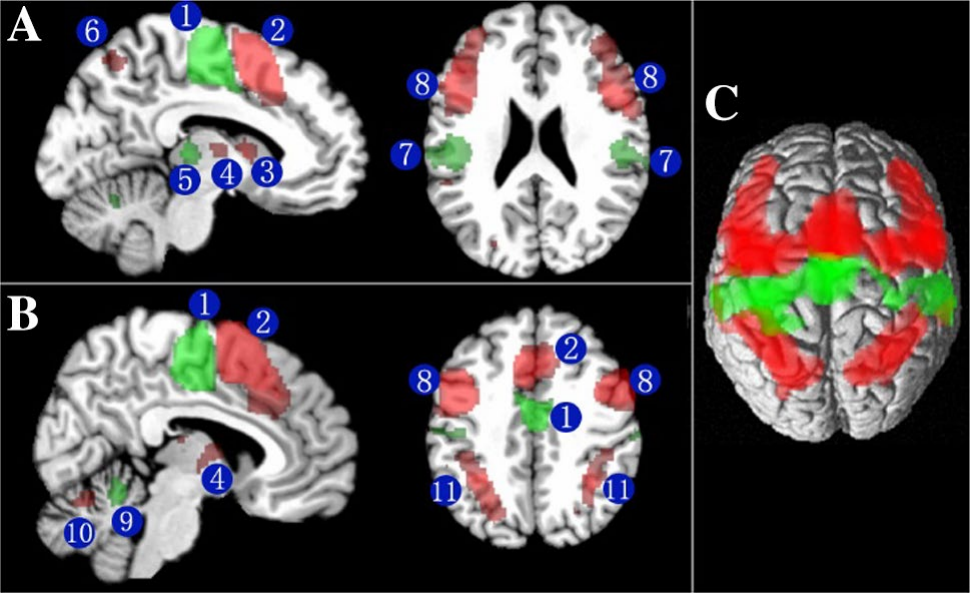
Contrast analysis of significant co-activation clusters for areas SMA and pre-SMA overlaid on the single-subject MNI template brain. The green colored regions are significantly stronger co-activated with area SMA. The red colored regions are significantly stronger co-activated with area pre-SMA.** a**,** b** Section through the MNI template showing the significant stronger co-activation clusters for area SMA and pre-SMA. Arabic numerals indicate the cluster locations with cytoarchitectonic informed anatomical labeling: 1 SMA; 2 pre-SMA; 3 caudate; 4 thalamus; 5 putamen; 6 superior parietal lobule; 7 superamarginal gyrus; 8 inferior frontal gyrus; 9 cerebellar Vemis; 10 cerebellums(VI); 11 inferior parietal lobule.** c** 3D rendering of the contrast of significant co-activation clusters for areas SMA and pre-SMA in MNI space. The anatomical localization of imaging results was determined by means of the SPM Anatomy Toolbox (Eickhoff et al. 2007)

The MPMs were used as seed regions for the MACM analysis using the BrainMap database. According to the coordinate-based meta-analysis of areas SMA and pre-SMA, 15 co-activation clusters for area SMA and 12 co-activation clusters for area pre-SMA were found in cortical and subcortical locations (Table 5). MACM analysis showed that both area SMA and pre-SMA were strongly co-activated with the precentral, supramarginal and superior frontal gyri, Rolandic operculum, thalamus, putamen and cerebellum. The contrast analysis of co-activation clusters between areas SMA and pre-SMA showed that the two areas have functionally dissociating connections. Area SMA was significantly more strongly co-activated with the precentral and supramarginal gyrus and caudal dorsal premotor cortex. Area pre-SMA, however, was significantly more strongly co-activated with the inferior parietal lobule, posterior parietal cortices and rostral dorsal premotor cortex (Fig. 8).

**Table 5:** Functional co-activation clusters for areas SMA and pre-SMA

Macroanatomical location	Hemisphere	Cytoarchitectonic area	Cluster size [voxels]	Anatomical MNI		
				*x*	*y*	*z*
Area SMA						
Posterior–medial frontal	L	Area 6mr/pre-SMA [39%]	450	− 4	− 4	56
Precentral Gyrus	L	Area 4a [58%]	202	− 38	− 24	58
Supramarginal gyrus	L	Area op1 [sii] [74%]	271	− 52	− 24	20
Putamen	L	Putamen (medial) [93%]	333	− 26	− 4	4
Superior frontal gyrus	L	Area 6d2 [46%]	196	− 26	− 4	60
IFG (p.opercularis)	L	Area 44 [41%]	321	− 48	6	28
Postcentral gyrus	L	Area 2 [68%]	393	− 36	− 40	54
Putamen	R	Putamen (medial) [83%]	282	24	2	4
Rolandic operculum	R	Area 44 [34%]	295	54	6	8
Superior frontal gyrus	R	Area 6d3 [71%]	162	26	− 4	56
Cerebellum (VI)	R	Lobule vi (hem) [94%]	451	20	− 54	− 22
Cerebellar vermis (4/5)	R	Lobule v (hem) [82%]	191	6	− 60	− 16
Supramarginal gyrus	R	Area PFcm (IPL) [53%]	121	60	− 30	26
Rolandic operculum	R	Area op1 [sii] [44%]	165	52	− 24	20
Cerebellum (VI)	L	Lobule VI (hem) [93%]	215	− 14	− 62	− 20
Pre-SMA						
Precentral gyrus	L	Area 44 [25%]	480	− 48	6	30
Precentral gyrus	L	Area 6d3 [38%]	245	− 28	− 4	56
Thalamus	L	Thal: Prefrontal [88%]	347	− 12	− 16	6
Thalamus	R	Thal: Prefrontal [84%]	310	12	− 16	6
IFG (p. Opercularis)	R	Area ifj2 [32%]	125	50	10	26
Putamen	L	Putamen (medial) [65%]	442	− 22	2	4
Inferior parietal lobule	R	Area hIP3 (IPS)	362	36	− 50	48
Superior parietal lobule	R	Area 7A (SPL) [30%]	248	18	− 66	54
Cerebellum (VI)	L	Lobule VI (Hem) [95%]	498	− 30	− 60	− 26
Inferior occipital gyrus	L	Area FG4 [58%]	175	− 44	− 60	− 14
Cerebellum (VI)	R	Lobule VI (Hem) [94%]	644	30	− 62	− 26
Superior temporal gyrus	R	Area TE2.2 [73%]	145	58	− 22	6

Cluster maxima assigned to the most probable cytoarchitectonic area when present in the SPM Anatomy Toolbox (Eickhhoff et al. 2005)

**Fig. 9:**
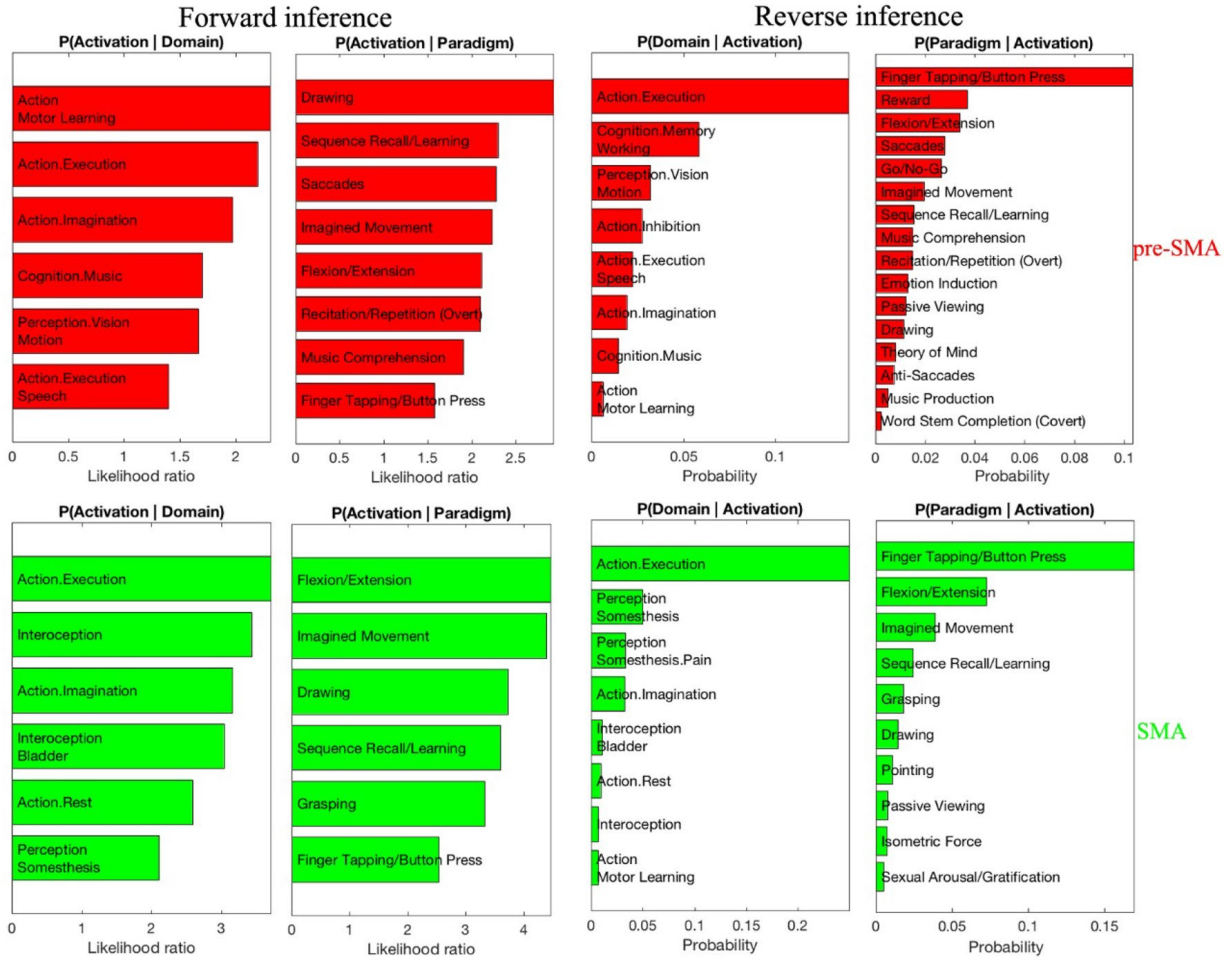
Significant functional clusters of area SMA (green color) and area pre-SMA (red color) with behavioral domains (top row) and paradigm classes (bottom row) of the BrainMap meta-data

Functional characterization according to the BrainMap meta-data was performed for area SMA and pre-SMA. We found areas SMA and pre-SMA to share some common behavioral domains (action execution and action imagination) and some paradigms (drawing, flexion/extension, imagined movement, and finger tapping/button press). Apart from that, area SMA was significantly related with domains such as interoception (and also more specifically with the domain associated with the bladder), and somesthetic perception. Area pre-SMA was correlated with domains such as action (motor learning, execution speech), cognition (in particular with the specific music domain), and the visual perception of motion. Moreover, the area pre-SMA was associated with tasks like sequence recall/learning, saccades and music comprehension. The contrast analysis of behavioral domains revealed that area SMA was more associated with action execution than area pre-SMA (Fig. 9). These results demonstrated that both areas SMA and pre-SMA were related to motor functions, but area pre-SMA was related to multiple functions beyond motor control such as perception, learning and cognitive perception.

## Discussion

In the present study, we applied a novel approach for 3D quantitative and statistically testable mapping to identify and differentiate areas SMA (SMA proper) and pre-SMA on the mesial surface of the hemisphere rostral to the primary motor cortex. Specifically, in stereotactic space the pre-SMA was localized rostral and the SMA caudal to the vertical plane through the anterior commissure. Furthermore, the different cytoarchitectonic patterns of the two areas were used to investigate if the two regions were part of different functional networks within the brain as evident from functional brain imaging. For that purpose, a meta-analysis of resting state functional imaging data was performed to demonstrate differential connectivity patterns. Furthermore, a meta-analysis of functional activation data classified according to different behavioural categories was employed to explore to what extent areas SMA and pre-SMA belonged to different functional networks. The combined findings were expected to provide evidence for a functional differentiation of human areas SMA and pre-SMA from the microscopical cytoarchitectonic to the macroscopical functional imaging level.

### Cytoarchitecture of areas SMA and pre-SMA

Agranular area SMA was characterized by a poor lamination, and densely packed large size pyramidal cells in the lower part of layer III. Agranular area pre-SMA presented a more differentiated laminar pattern and a more pronounced layer V. These findings are in accordance with previous cytoarchitectonic studies (Zilles et al. 1995, 1996; Vorobiev et al. 1998). We were not able, however, to confirm Vorobiev’s (1998) subdivision of SMA proper into two parts: SMA caudal and SMA rostral—no significant borders were detected within SMA. The photomicrographs of SMA caudal in Vorobiev’s study might depict the transition between the rostral portion of BA4 and the caudal portion of his area 6 (SMA proper), since they showed large pyramidal cells in layer V with the size which was between that of Betz cells and large sized cells of layer V in area SMA. Therefore, the so-called caudal SMA may be the transition between BA4 and area SMA, and was not part of the present study.

Subsequent analysis of profiles based on their subdivision into 10 bins allowed to identify differences between SMA and pre-SMA at bins 4 and 5 (40%-50%), which roughly corresponded to the position of lower layer III. This suggests that the most prominent differences between areas SMA and pre-SMA were differences of cell packing density in layer IIIc.

Importantly, the cytoarchitectonic border between areas SMA and pre-SMA was located close to the vertical plane through the anterior commissure (*Y* = 0), which is in good agreement with earlier cytoarchitectonic data provided by Zilles et al. (1996) and in-vivo data as delineated by diffusion tractography (Johansen-Berg et al. 2004). Although an inherent limitation exists in anatomical accuracy of brain connections derived from diffusion tractography (Thomas et al. 2014), this concordance between the two different techniques performed on independent samples is noteworthy considering that this provides an approach to in-vivo investigation of human brain organization, which shows a good congruency with cytoarchitectonic mapping in postmortem samples.

Nevertheless, there was a notble difference between the cytoarchitectonic analysis and the MACM analysis, as the co-activation pattern lolacized the pre-SMA more anterior than the cytoarchitectonically delineated pre-SMA. This may result from the fact that the saccade task was related to the SMA as derived from functional co-activation (Eickhoff et al. 2011), but related to the pre-SMA as derived from cytoarchitecture analysis. Such a discrepancy may be accounted for by the fact that cytoarchitecture reflects the inner organization of cortical areas which is related but not equal to the functional properties of these regions.

### From architectonical to functional gradation

Based on earlier observations of Vogt (1919) and of Brockhaus (Brockhaus 1940), Sanides introduced the term “gradation” and “Gradationsprinzip” (gradation principle) to explain the existence of structural differentiation “streams” in the cerebral cortex (Sanides 1962, 1964). In fact, our present observer-independent procedures can detect such structural differentiation “streams” and, thereby, provide insight into the underlying evolution principles of regional differentiation (Henssen et al. 2016; Zilles and Amunts 2012). According to the gradation principle, the mesial frontal motor cortex originates from the posterior precentral cortex (Sanides 1962, 1964). In our study, we found a gradation stream in the sense that the cell size in layer V relatively increased when moving from caudal area SMA to rostral area pre-SMA. Moreover, we found that from areas SMA to pre-SMA, from areas 24d to 24c, from areas PMdc to PMdr, they shared some common changes of cytoarchitectonic characterizations: The relative cell density in layer II decreased; the relative cell density and cell size in layer IIIc decreased; the cell density in layer V increased. These findings in these areas are in accordance with the notion that cortical areas do not simply form a mosaic, but are hierarchically organized (Amunts and Zilles 2012). Thus, our findings suggest that gradation streams not only exist between large regions (e.g., from area 4 to area 6), but also between subareas (e.g., from area SMA to area pre-SMA).

The architectonic gradation is likely to be accompanied by a functional gradation across the areas. According to the behavioral domain and paradigm class-based analysis, we noted that area pre-SMA and area SMA are both involved in sensorimotor integration for action generation. The more posteriorly located primary motor cortex plays an important role in the execution of body movement (Hlustik et al. 2001; Schieber 2001). Interestingly, electric stimulation of the primary motor cortex, of pre-SMA, SMA resulted in the induction of movements, although it was more difficult to do so from pre-SMA than from SMA than from the primary motor cortex (Luppino et al. 1991). Moreover, our functional meta-analysis revealed that area SMA was closely linked to movement execution, while the pre-SMA was concerned with higher movement control mechanisms such as in procedural learning and language production (Ikeda et al. 1999; Seitz et al. 2006; Rochas et al. 2013). Thus, when moving from the primary motor cortex through SMA and into pre-SMA, the areas’ involvement in movement execution became more remote. This suggests that in the human brain at least some functions are associated with subtle gradations in parallel to the cytoarchitectonic gradation changes rather than in a mosaic-like fashion.

### Functional connectivity of areas SMA and pre-SMA

The coordinate-based meta-analytic connectivity modeling analysis allowed us to identify the brain areas that constituted functional neural networks for areas SMA and pre-SMA. We found that both of them accommodate extensive cortico-subcortical circuits involving the precentral gyrus, supramarginal gyrus, superior frontal gyrus, Rolandic operculum, thalamus, putamen and cerebellum. However, the anterior cluster corresponding to area pre-SMA derived showed significantly higher co-activation probabilities with inferior frontal and posterior parietal cortices. By contrast, the posterior cluster was more strongly co-activated with precentral gyrus, and caudal dorsal premotor cortex. Some of these co-activations resulted from true anatomical connectivity. For example, areas SMA and pre-SMA project to the putamen and cerebellum (Alexander et al. 1986; Akkal et al. 2007). Likewise, SMA and pre-SMA are connected with the precentral gyrus and with more anterior portions of the superior frontal gyrus (Luppino et al. 1993). However, areas SMA and pre-SMA also were found to have differential connections. For example, area SMA had a strong association with the putamen, while area pre-SMA was more strongly related to the caudate nucleus. This was in accordance with the results of an earlier fibre tracking study (Alexander et al. 1986). It must be emphasized, however, that functional connectivity does not imply a direct anatomical connection between the respective brain regions (Eickhoff and Grefkes 2011). For example, the co-activation patterns between areas SMA, pre-SMA and the Rolandic operculum are functional rather than reflecting direct anatomical connections among them.

Despite theis congruency, some differences were found when anatomical were taken into consideration. In diffusion tensor imaging the area pre-SMA had dense anatomical connections to the medial parietal cortex (Johansen-Berg et al. 2004), which were not apparent in the present study. Moreover, MACM showed that areas SMA and pre-SMA had significant connections with the cerebellum, which were not presented in diffusion tractography (Johansen-Berg et al. 2004). This may be explained by the different approaches employed in the two studies. The anatomical connectivity and the functional connectivity are conceptually different: the former is a task-independent property, while the latter is a task-dependent property (Eickhoff et al. 2011).

### Comparison to resting state functional connectivity

The human mesial frontal cortex has also been parcellated based on the similarity of functional connections using resting state functional MRI (Kim et al. 2010). The vertical plane through the anterior commissure, a reliable macroanatomical landmark associated with the cytoarchitectonically identified border between areas SMA and pre-SMA (present observations; Zilles et al. 1996), also constituted the border between the anterior and posterior clusters derived from resting state functional MRI (Kim et al. 2010). It was reported that the functional networks in “rest” showed a close correspondence with the functional networks in explicit “activation” (Smith et al. 2009; Simonyan and Fuertinger 2015).

### Primate data on ares SMA and pre-SMA

Areas SMA and pre-SMA were identified and differentiated from each other also in non-human primates using cytoarchitectonic, connectivity and stimulation studies. Cytoarchitectonic studies revealed in the agranular mesial frontal cortex two distinct areas one of which was labeled F3 corresponding to SMA and the other F6 corresponding to pre-SMA (Matelli et al. 1991). Ventrally adjacent four distint areas in the cingulate gyrus and on the lateral convexity a number of subareas in premotor cortex were identified (Matelli et al. 1991). It was found that SMA is richly linked with motor cortex, posterior premotor cortex and dorsal cingulate and parietal cortical areas, while pre-SMA receives input from the anterior premotor cortex, midfrontal cortex and midcingulate area 24c but has no connections to motor cortex (Luppino et al. 1993). In addition, SMA receives about half of the callosal input from its counterpart in the other hemisphere, whereas pre-SMA receives input preferentially from dorsal and ventral premotor cortex, the cingulate motor area, and prefrontal cortex (Liu et al. 2002). In addition, there are strong projections from subcortical brain structures including the pallidum, thalamus, and the cerebellum via thalamus to the SMA (Rouiller et al. 1994). Both areas have efferent connections to subcortical loactions with segregated corticostriatal and corticosubthalamic input zones (Inase et al. 1999).

These anatomical differences appear to accommodate functional differences among these two cortical areas, as neuronal activity differed between SMA and pre-SMA in relation to instruction and execution of movmements. For example, in a visuomotor task neural activity in SMA was related to use of the right or left arm, while activity in pre-SMA was related to location of the target (Hoshi and Tanji 2004). There is some indication of somatotopic organization of the SMA as evident from microstimulation mapping (Mitz and Wise 1987; Luppino et al. 1991) which may be related to strong corticospinal projections from the SMA (Galea and Darian-Smith 1994). In contrast, microstimulation of the pre-SMA frequently resulted in slow forelimb movements that were similar to natural movements (Luppino et al. 1991). Furthermore, pre-SMA seems to code more complex aspects of movement patterns as pre-movement activity in pre-SMA was found to be selective to the second-next movement before initiation of the first movement (Nakajima et al. 2009). Moreover, in sequential motor tasks performed from memory neuronal activity in pre-SMA resembled a binary-code which was uncommon in SMA (Shima and Tanji 2006).

### Functional distinction of mesial frontal cortex and neighboring areas

Adjacent to pre-SMA, but not SMA, there are a number of neighboring areas referred to as anterior midcingulate cortex (aMCC), dorsomedial prefrontal cortex (dmPFC), and PMd which also were characterized by multimodal mapping similarly as in this study. The functionally defined region within the aMCC (Hoffstaedter et al. 2014) showed largely overlapping co-activation clusters in lateral premotor cortex, Rolandic operculum, putamen, inferior parietal lobule, cerebellum and thalamus similarly to the present study but not with the supramarginal gyrus, superior temporal gyrus, superior parietal lobule and inferior occipital gyrus. In contrast, the dorsolateral prefrontal cortex and intraparietal sulcus were more likely co-activated with that functionally defined region within aMCC (Hoffstaedter et al. 2014) than in our study. It is possible that the difference was due to the role of aMCC in intentional movement initiation involving pain and negative affect (Shackman et al. 2011; Hoffstaedter et al. 2013; Tolomeo et al. 2016).

In addition, it was noted that mesial frontal cortex and dmPFC showed overlapping functional connectivity with inferior parietal cortex, inferior frontal gyrus and prefrontal cortex. However, the dmPFC was co-activated specifically with clusters involving emotion like amygdala and hippocampus (Eickhoff et al. 2016). In contrast, the mesial frontal cortex was more strongly co-activated with the precentral gyrus, supramarginal gyrus, lateral premotor cortex, superior temporal gyrus, superior parietal lobule, putamen, cerebellum and thalamus. Recently, the dmPFC was related to emotion and social cognition (Schurz et al. 2014; Wagner et al. 2016) which was not found in the functional coding analysis of mesial frontal cortex in present study.

The laterally neighboring area of mesial frontal cortex, PMd, which has been investigated using the same approach as the present study was found to be co-activated with the precentral gyrus, thalamus, putamen and cerebellum. However, regionally specific co-activation clusters suggested that PMd was more likely to co-activate with dorsal prefrontal cortex (Benjamin et al. 2016). In addition, the mesial frontal cortex connected with cerebellum at lobule VI, while PMd connected with dorsal surface of cerebellum (Benjamin et al. 2016). Both PMd and mesial frontal cortex were related to action (execution and imagination) and interoception as well as memory and movement sequence processing (Pastor-Bernier et al. 2012; Solopchuk et al. 2016).

## Conclusions

In this anatomical and multimodal imaging approach based on an observer-independent methods for identifying borders of cortical areas, we were able to demonstrate that the dorsal mesial frontal cortex comprised of two structurally and functionally heterogeneous areas: area SMA and pre-SMA supporting and extending earlier observations in humans and non-human primates (Picard and Strick 1996). The probabilistic maps of area SMA and pre-SMA in standard MNI space are steps toward a complete map of the human cerebral cortex based on an observer-independent quantitative and statistically testable cytoarchitectonic parcellation approach. Using the maximum probability maps of area SMA and pre-SMA as seed regions for a coordinate-based neuroimaging meta-analysis, we were able to describe also a functional segregation between areas SMA and pre-SMA. Although, our results further support the notion that the integration of different methodological approaches distinguishing cortical modules is necessary to understand the link between brain structure and function (Amunts and Zilles 2015), future studies are needed to account for the diversity and integration of human brain function.

## Electronic supplementary material

Below is the link to the electronic supplementary material.

Supplementary material 1 (DOCX 4442 KB)
