# Supplementary material for: Cytoarchitecture, probability maps, and functions of the human supplementary and pre-supplementary motor areas
Source: Brain Struct Funct. 2018 Sep 5;223(9):4169–86. doi: 10.1007/s00429-018-1738-6 (PMC6267244; doi:10.1007/s00429-018-1738-6)
Supplement: Supplementary file 1 — Supplementary material 1 (DOCX 4442 KB) [file 429_2018_1738_MOESM1_ESM.docx]

# Supplementary Figures

Supplementary Fig. 1. Posterior-to-anterior sequences of eight coronal histological sections through the left and right hemispheres of cases 01 and 06. Areas SMA and pre-SMA are shown by red and blue color, respectively.


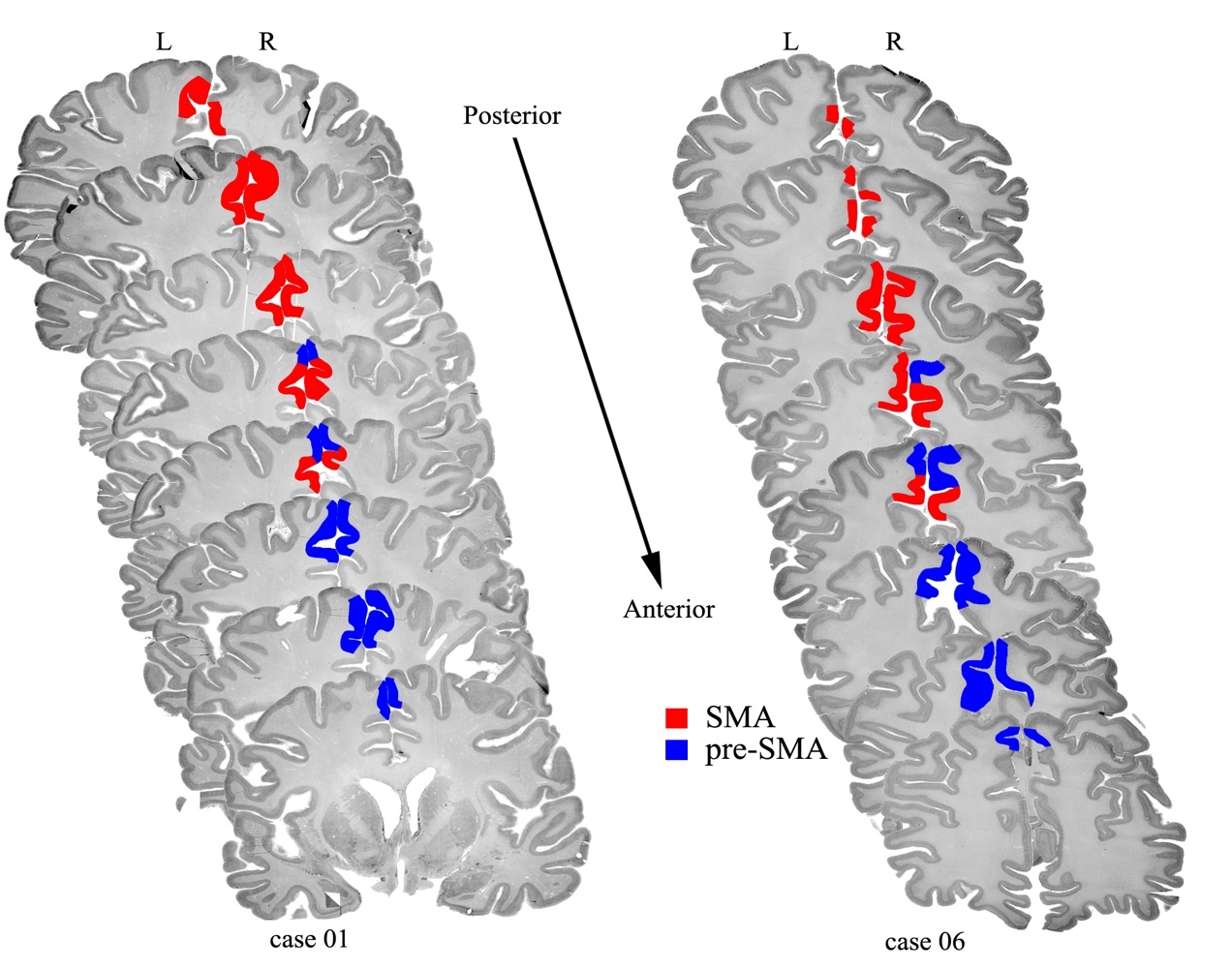


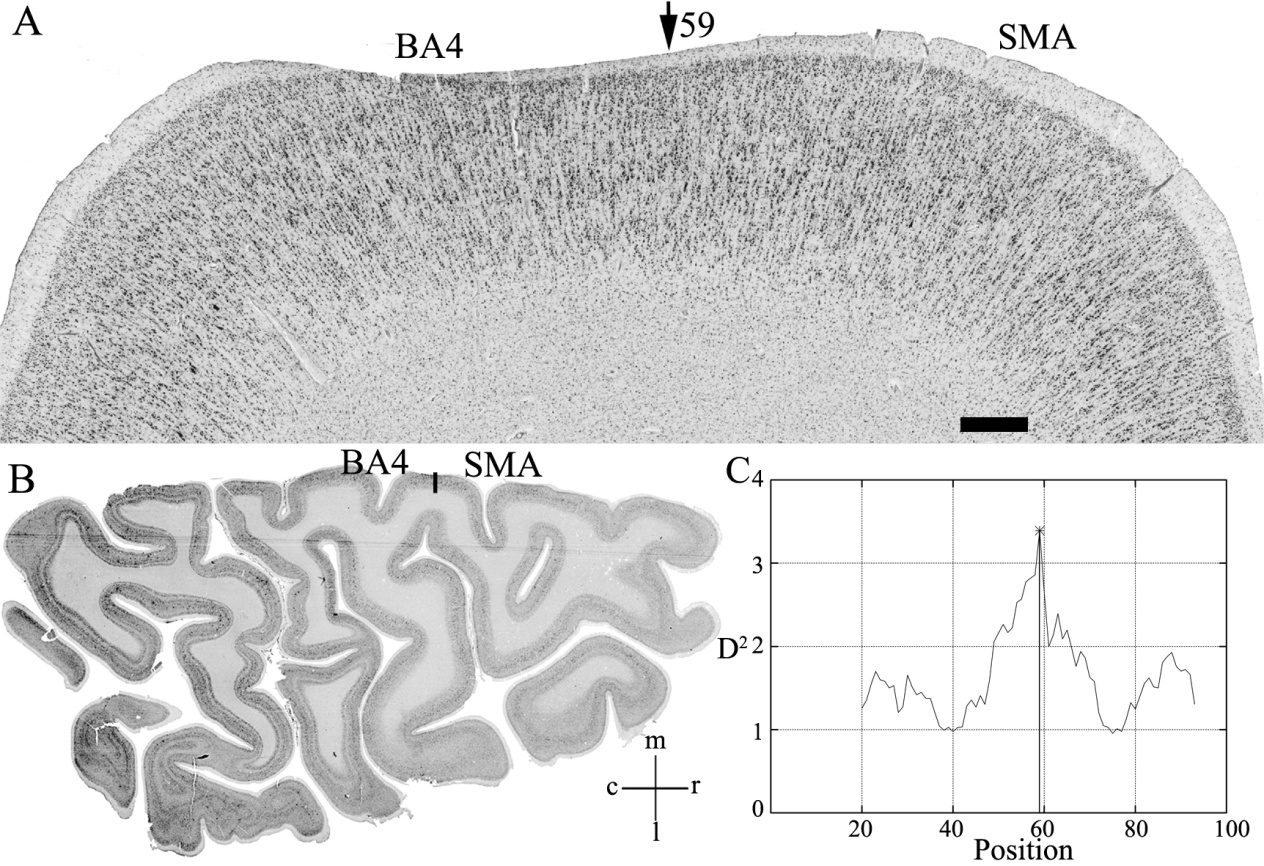


Supplementary Fig. 2. Caudal border of area SMA with Brodmann’s area 4 (BA4). A: photomicrograph of a section from case 17 showing the border between areas SMA and BA4 (arrowhead). The arrowhead indicates the position of profile#59. B: location of the region of interest. C: significant maximum at position 59 indicates the border between areas SMA and BA4. m=mesial, l=lateral, r=rostral, c=caudal. Scale bar = 0.5 mm.


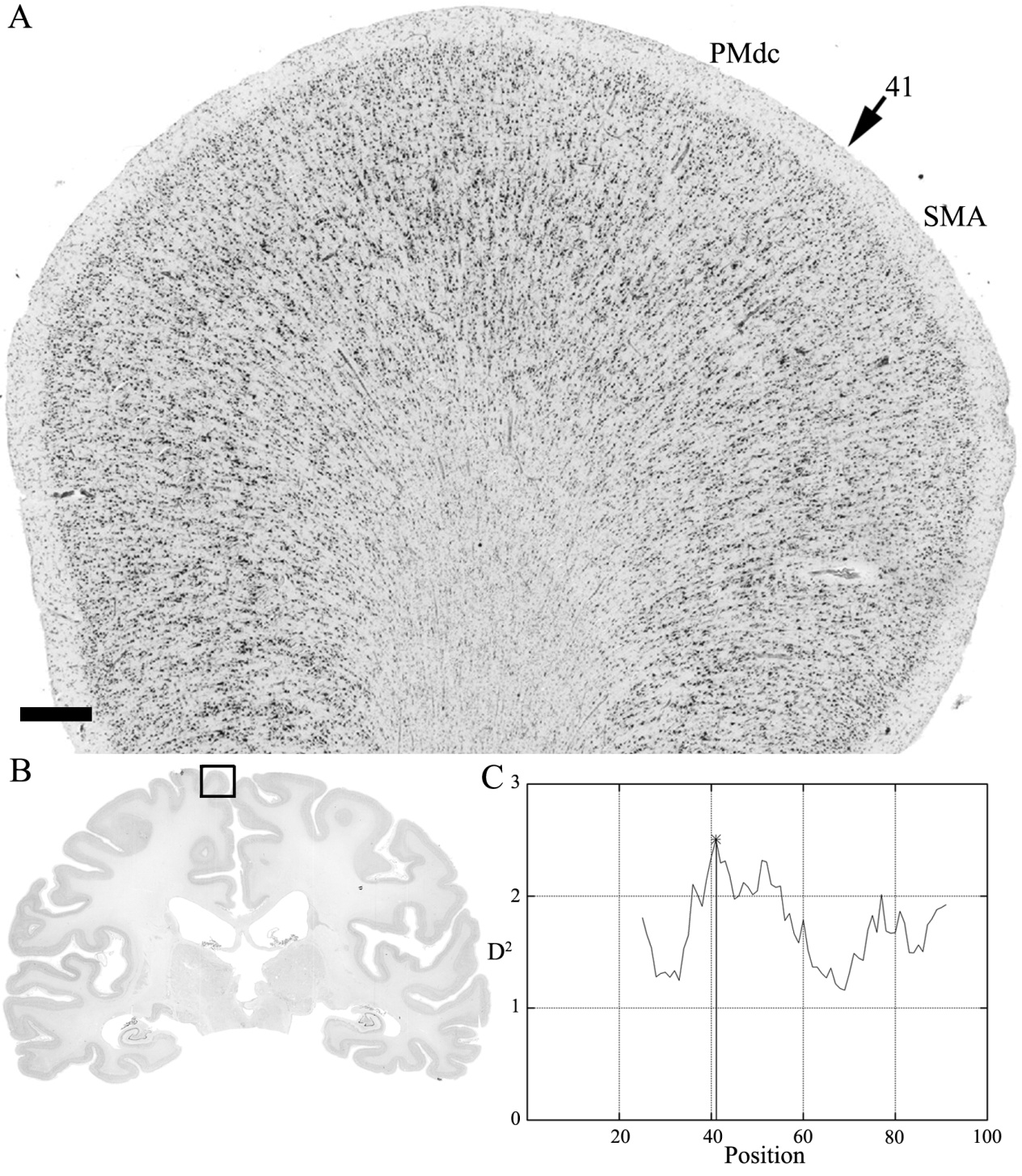


Supplementary Fig. 3. Dorsal caudal border of area SMA with PMdc. A: photomicrograph of a section from case 4 showing the border between areas SMA and PMdc (arrowhead) at the position of profiles 41. Note the darker layer III and layer V and well demarcated layer VI in PMdc. B: location of the region of interest. C: significant maximum at position 41 corresponding with figure A indicates the border between areas SMA and PMdc. PMdc, premotor dorsal caudal. Scale bar = 0.5 mm.


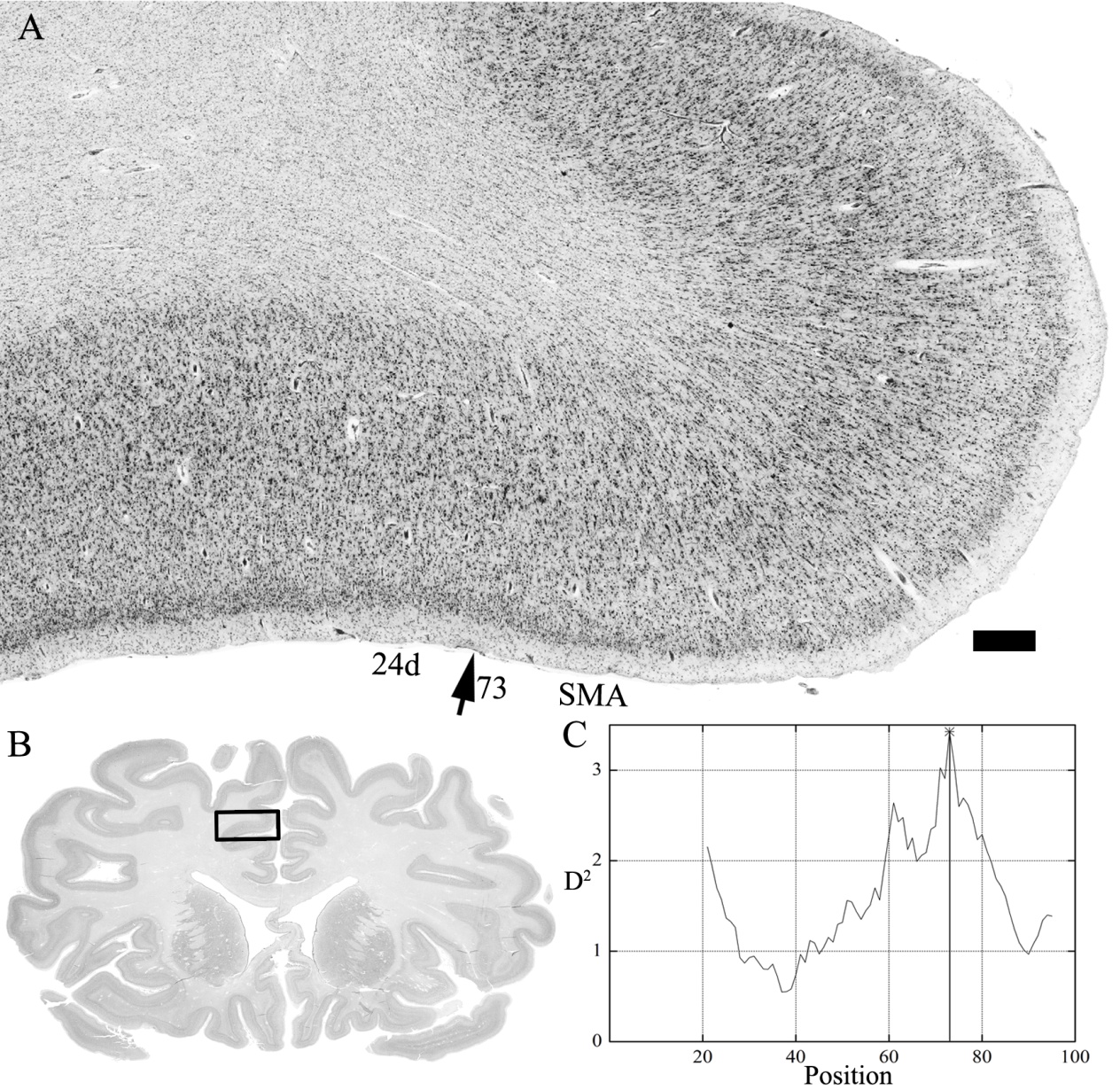


Supplementary Fig. 4. Ventral border of area SMA with area 24d. A: Photomicrograph of a section from case 10 showing the border between areas SMA and 24d (arrowhead). The arrow indicated positions of profiles 73. B: Location of the region of interest. C: Significant maximum at position 73 indicates the border between areas SMA and 24d. Scale bar = 0.5 mm.


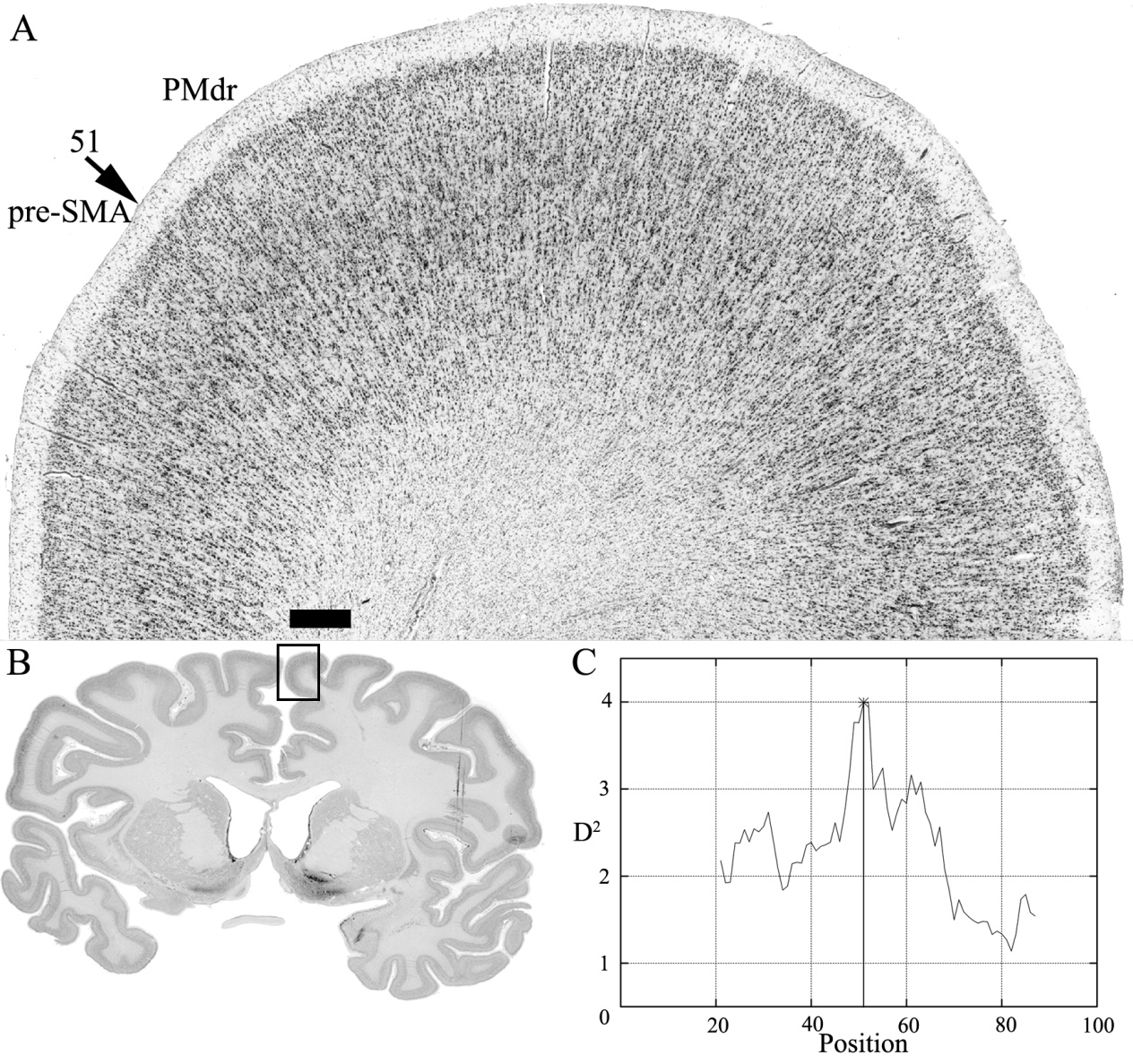


Supplementary Fig. 5. Dorsal rostral border of area pre-SMA with PMdr. A: photomicrograph of a section from case 12 showing the border between areas pre-SMA and PMdr (arrowhead). The arrowhead indicates position of profiles 51. Note the darker layer V and higher overall cell density in PMdr. B: location of the region of interest. C: significant maximum at position 51 corresponding with figure A indicates the border between areas pre-SMA and PMdr. PMdr, premotor dorsal rostral. Scale bar = 0.5 mm.


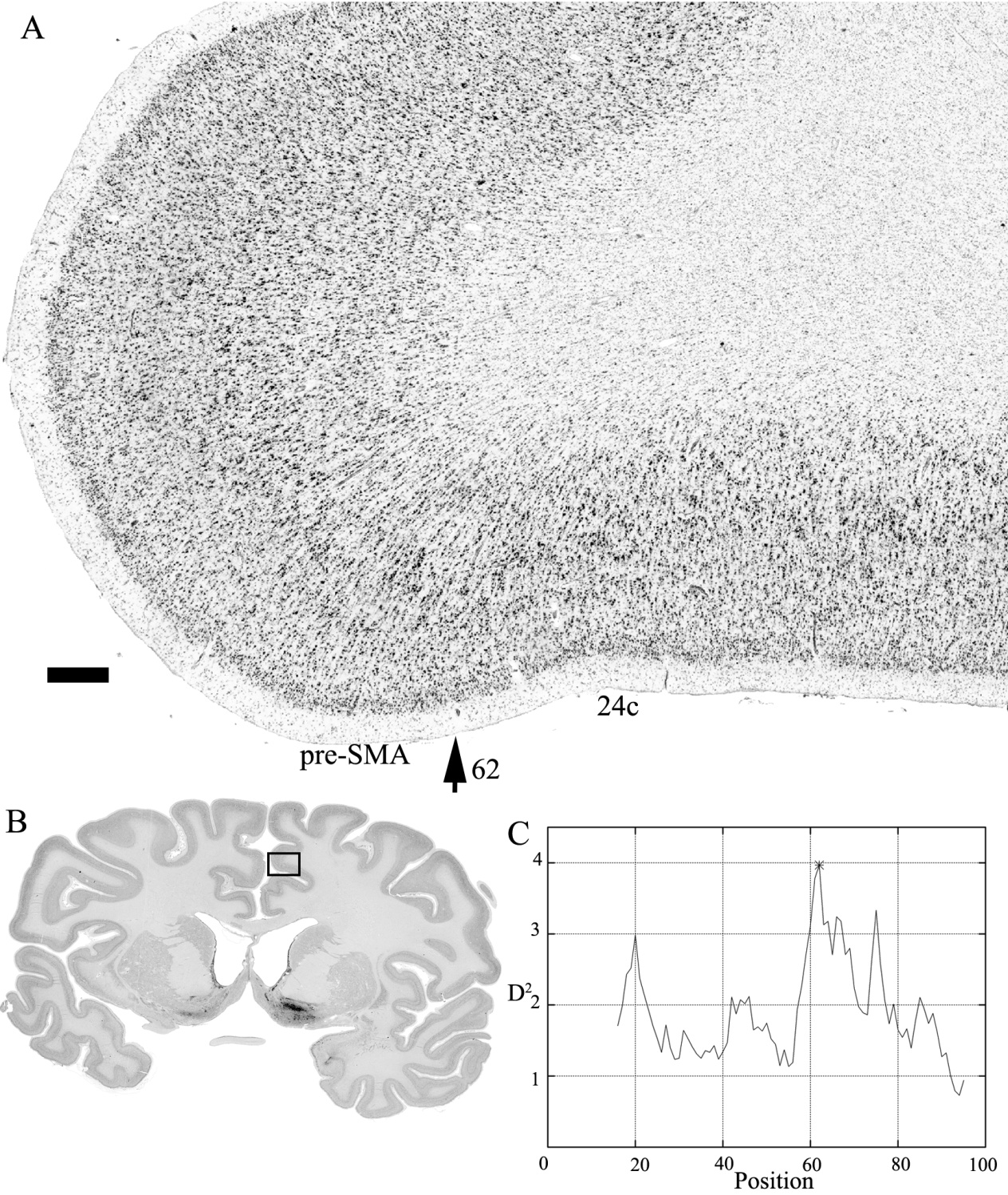


Supplementary Fig. 6. Ventral border of area pre-SMA with area 24c. A: Photomicrograph of a section from case 12 showing the border between areas SMA and 24c (arrowhead). The arrow indicated positions of profiles 62. B: Location of the region of interest. C: Significant maximum at position 62 indicates the border between areas pre-SMA and 24c. Scale bar = 0.5 mm.


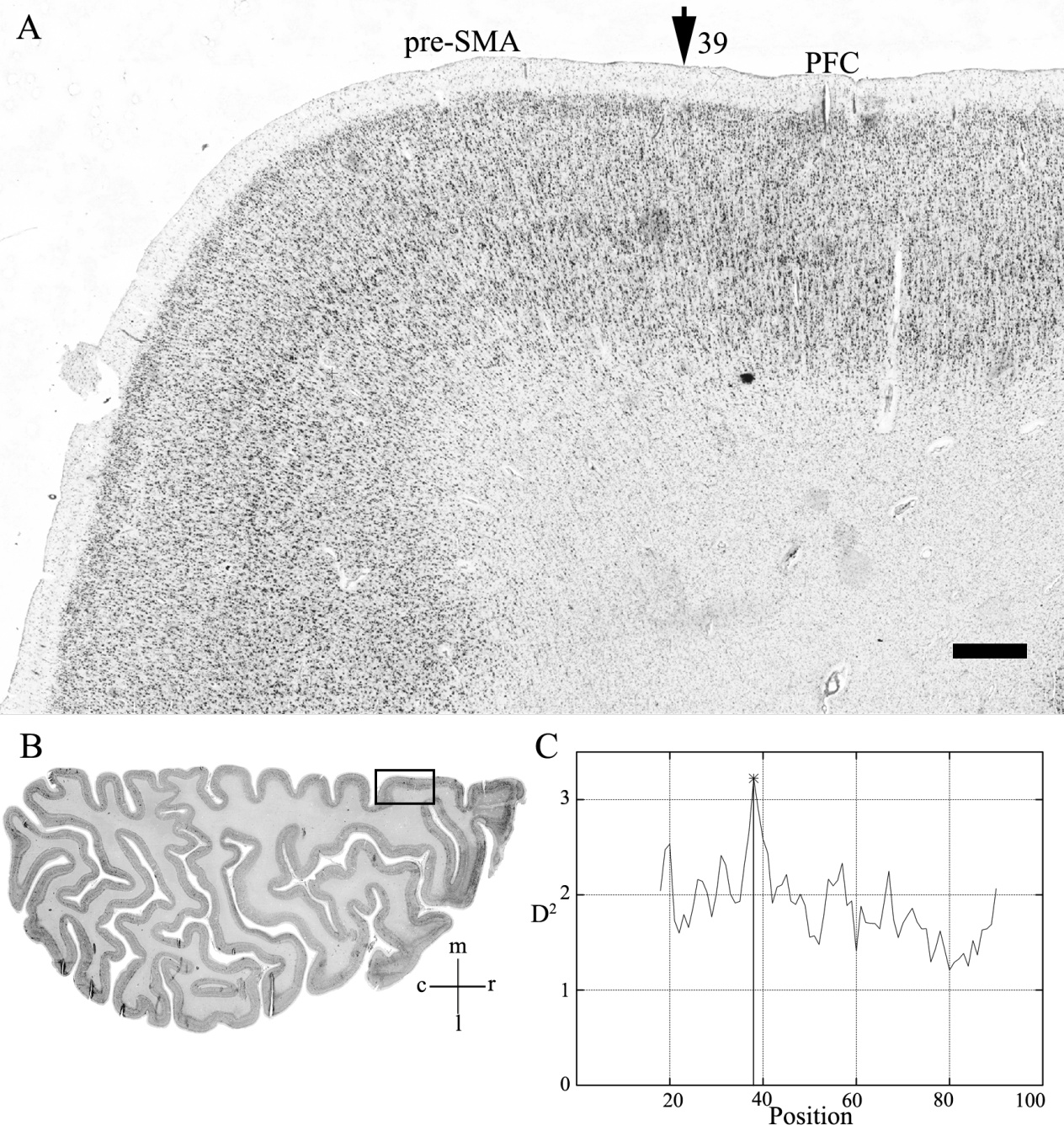


Supplementary Fig. 7. Rostral border of area pre-SMA with area PFC. A: Photomicrograph of a horizontal section from case 17 showing the border between areas pre-SMA and PFC (arrowhead). The arrow indicated positions of profiles 39. B: Location of the region of interest. C: Significant maximum at position 39 indicates the border between areas pre-SMA and PFC. PFC, prefrontal cortex. Scale bar = 0.5 mm, m=mesial, l=lateral, r=rostral, c=caudal.
